# The impact of long-term moderate level of vaccination coverage for epidemiology of varicella in Lu'an, China: should we change immunisation strategy now?

**DOI:** 10.1017/S0950268820000667

**Published:** 2020-03-13

**Authors:** Wei Qin, Xiangmei Meng, Liang Zhang, Yao Wang, Xiaokang Xu, Kaichun Li, Shaoyu Xie

**Affiliations:** 1 Department of Expanded Program on Immunization, Lu'an Municipal Center for Disease Control and Prevention, Lu'an 207008, Anhui, China; 2 Department of Emergency, Lu'an Affiliated Hospital of Anhui Medical University, Lu'an People's Hospital, Lu'an 237008, Anhui, China; 3 Jin'an District Center for Disease Control and Prevention, Lu'an 237008, Anhui, China

**Keywords:** Epidemiology, vaccination coverage, vaccine effectiveness, varicella

## Abstract

As China implements the voluntary vaccination programme of one-dose of varicella vaccine (VarV) for decades, robust estimates of the impact of voluntary vaccination era on epidemiology of varicella are needed. We estimated the vaccination coverage (VC) of VarV by using surveillance data on immunisation. The descriptive epidemiological method was used to describe the changing epidemiology of varicella from 2007 to 2018. The screening method was used to estimate the vaccine effectiveness (VE) of VarV. The overall VC for VarV was 71.7%, ranged from 47.7% to 79.5% among 2008–2017 birth cohorts. In total, 16 660 varicella cases were reported during 2007–2018, the incidence increased from 10.0 cases per 100 000 population in 2007 to 65.2 cases per 100 000 population in 2018. A shift in age group of varicella was observed since 2012, with the age increased from 5–9 years to 10–14 years. The overall VE was 79.9%, and the VE increased from 60.1% in 2008 birth cohort to 96.2% in 2017 birth cohort. We found that the overall VE for VarV is moderate, but appears highly effective within 5 years after vaccination. In addition, a shift varicella infection to older ages has occurred at the long-term moderate level VC of one-dose VarV. Therefore, to contain the incidence of varicella and prevent any potential shift to older ages, the introduction of VarV into routine immunisation programme is likely needed in Lu'an.

## Introduction

Varicella is highly contagious disease caused by varicella-zoster virus primary infection and endemic to all countries worldwide. Although varicella is usually a benign disease in children, it may cause complications, and the indirect burden may be underestimated due to the high number of cases and parents taking time off to look after their children [1]. Similar to many other vaccine preventable diseases, active immunisation is considered one of the most effective intervention for varicella. The live attenuated Oka strain varicella vaccine (VarV) was first developed in 1974 by Takahashi in Japan and has turned out to be safe and effective at preventing varicella [2]. A meta-analysis reported that the vaccine effectiveness (VE) against all varicella for one-dose and two-dose VarV was estimated at 81% and 92%, respectively [3]. Although there are only 36 countries and regions that have introduced VarV for routine immunisation, few of them implemented a two-dose schedule currently [4]. This may due to concerns about cost-effectiveness, a shift in disease to older ages and an increase in herpes zoster in the elderly [1, 4].

Although studies showed that countries implementing a universal varicella vaccination have experienced a dramatic decline in varicella incidence and deaths [5–7], varicella outbreaks and breakthrough varicella still occur. Therefore, a routine two-dose schedule was recommended in the USA and Germany [8, 9]. In China, VarV was first licensed in 1998 for using as one-dose schedule in the private sector targeting children aged at least 12 months. However, the incidence of varicella showed an increasing trend in China from 2005 to 2015 [10]. A meta-analysis to evaluate varicella vaccination coverage (VC) among Chinese children found an obvious disparity among different provinces, where the pooled coverage was 97.3% in the eastern region and 40.8% in the central and western regions [11]. The disparity of VC between the regions in China might be caused by differing levels of economic development [11]. Frustratingly, even in the eastern region with high coverage, the decline of varicella morbidity was not observed until a second dose was recommended [12]. Although two-dose schedule was not recommended nationally in China, some developed provinces which have recommended a second dose at 4 years of age or older have experienced a substantial decline in varicella incidence [12, 13]. However, provinces in the central and western regions such as Anhui which have not recommended a two-dose schedule, the impact of the sustaining low or moderate level of VC on varicella should be evaluated.

In 2018, a total of six varicella outbreaks with high breakthrough varicella occurrence rate (range: 4–32%) occurred in junior high schools in Lu'an, sparking local public health authority's concerns on the shift in varicella to older ages and the VE for VarV. Therefore, we used surveillance data to describe the changing epidemiology of varicella during 12 years of implementation of the one-dose voluntary vaccination strategy in Lu'an, and to evaluate the VC and the VE of VarV among 2008–2017 birth cohorts.

## Materials and methods

### Setting

Lu'an city is located in the central region of China, adjacent to Henan Province and Hubei Province. It has the highest total area of 15 451 square kilometres in Anhui Province and about 5.88 million permanent residents.

### Study design and data sources

This is a three-stage study. In the first stage of the study, we aimed to evaluate the VC of VarV and compare the coverage of VarV with the coverage of the second dose of measles containing vaccine (MCV_2_), which had been introduced in Expanded Program on Immunization (EPI) since 2008. The reasons why we chose MCV_2_ to compare with the VC of VarV are as follows: (1) since the age of administering MCV_2_ was almost the same as that of VarV vaccination (18 months *vs.* 12 months), the number of target children may not fluctuate too much, (2) to compare the disparity between the VC between EPI vaccine (since 2008) and non-EPI vaccine. Data on VarV were generated and collected within Anhui Immunization Information Management System (AIIMS). The AIIMS is an internet-based management platform maintaining immunisation data for children aged <7 years living in Anhui province. All vaccination clinics in Anhui have been required to install a client application software, and a database deployed in Anhui Center for Disease Control and Prevention since 2007. When a child receives a vaccination, vaccination clinics workers send basic demographic characteristics and information about the vaccine used to the database. Reported information included name, date of birth, gender, name of parents, address, date and dose of vaccination, vaccine types and phone number. If migrant children living in Anhui for more than 3 months, historical immunisation information recorded in vaccination certificates will be entered into the AIIMS. Children who were deceased, moved or went elsewhere (i.e. not living in Anhui for more than three months) were excluded in the AIIMS. Demographic information and vaccination records of 2008–2017 birth cohorts were extracted from the AIIMS database on 1 July 2019. In the second stage of the study, we aimed to describe the varicella epidemiology characteristics in Lu'an, China, 2007–2018. Data on varicella cases and incidence in this stage were derived from *China Information System for Disease Control and Prevention* (CISDCP) which was an internet-based, passive infectious disease surveillance system in China. Although varicella is not a notifiable disease, it is an infectious disease under intensive surveillance. Therefore, the cases diagnosed by doctor should be reported in the CISDCP since 2005 in China. The main contents of collecting information included name, date of birth, gender, name of parents, date of the onset, address, etc. All data were collected from CISDCP up to 1 July 2019. In the third stage of the study, we aimed to assess the VE for VarV for 2008–2017 birth cohorts. We estimated VE against varicella of any severity in children using the screening method [14] as follows: VE = 1 − [(PCV/1 − PCV)(1 − PPV/PPV)], where PCV and PPV were the proportion of cases vaccinated and proportion of total population vaccinated, respectively. Moreover, 95% confidence intervals (CIs) were calculated by using the following formula: VE = 1 − (incidence rate_vaccinated_/incidence rate_unvaccinated_), where the ratio between the incidence rates among the vaccinated and unvaccinated individuals corresponds to a relative risk [15]. Data in this stage consisted of three parts: (1) extraction of varicella vaccination information in children born in 2008–2017 birth cohorts from AIIMS, (2) extraction of varicella cases that occurred in children born in 2008–2017 birth cohorts from CISDCP and (3) verify their vaccination status *via* matching names, date of birth, name of parents or address in the AIIMS. Those cases who could not be verified vaccination record were excluded from the cohorts.

### Definitions

The VC of varicella was defined as the proportion of children who received at least one dose of VarV, while the VC of MCV_2_ was defined as the proportion of children who received the second dose of MCV. Breakthrough varicella was defined as varicella infection occurring more than 42 days after the vaccination. A varicella outbreak was defined as 10 or more new cases of varicella epidemiologically linked to a common setting and occurred within 1 week.

### Data analyses

Data cleaning and descriptive epidemiology data analysis were performed by using Microsoft Excel 2016. Trends of incidence in different birth cohorts, coverage rates between VarV and MCV were compared by the chi-square test. All statistical analyses were performed using Epi Info™ software (version 7.2). Two-sided *P* values were reported to be statistically significant at <0.05.

## Results

### Estimated coverage for VarV and MCV_2_

A total of 432 560 children aged from 1.5–10.5 years (2008–2017 birth cohorts) were enrolled in our study. As shown in Table 1, there were some fluctuations in VC for VarV_1_ among 2008–2017 birth cohorts, with the highest VC occurring in 2012 birth cohort (79.5%) and the lowest VC occurring in 2008 birth cohort (47.7%). Compared with 2008–2009 birth cohorts, an increase in VC occurred in 2010–2017 birth cohorts, maintaining annual VC between 70.4% and 79.5%. A very low VC for VarV_2_ was observed among all birth cohorts, ranging from 0.03% to 3.3%. The VC for VarV_1_ among the 2008–2017 birth cohorts was significantly lower than that of MCV_2_ (94.9–98.0%).

**Table 1.** Estimated VC for VarV and MCV_2_ by birth cohort in Lu'an, Anhui, China, 2008–2017

Birth cohort	Population	Coverage of the VarV				Coverage of the MCV_2_	
		One-dose vaccinated	%	Two-dose vaccinated	%	Vaccinated	%
2008	46 870	22 343	47.7	619	1.3	44 461	94.9
2009	45 161	28 483	63.1	858	1.9	43 340	96.0
2010	43 663	30 728	70.4	1011	2.3	42 238	96.7
2011	42 866	32 589	76.0	1275	3.0	42 013	98.0
2012	43 718	34 762	79.5	1455	3.3	41 645	95.3
2013	40 702	31 977	78.6	1192	2.9	39 011	95.8
2014	41 222	32 674	79.3	1074	2.6	39 902	96.8
2015	40 821	31 157	76.3	525	1.3	38 844	95.2
2016	44 205	34 223	77.4	147	0.3	43 057	97.4
2017	43 332	31 113	71.8	14	0.03	41 549	95.9
Total	432 560	310 049	71.7	8170	1.89	416 060	96.2

### Changing epidemiology of varicella in Lu'an

**Fig. 1.**
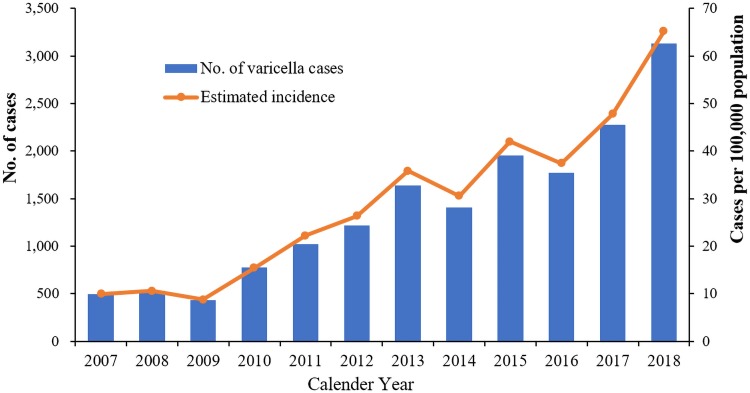
Number of cases and estimated incidence of varicella in Lu'an, Anhui, China, 2007–2018.

During 2007–2018, a total of 16 660 varicella cases were reported through CISDCP in Lu'an, Anhui, China. As shown in Figure 1, the annual number of reported cases increased 527%, from 499 in 2007 to 3130 in 2018; estimated incidence increased from 10.0 cases per 100 000 population to 65.2 cases per 100 000 population (*P* < 0.001). There was only one varicella death case reported. Of the 16 660 cases, 10 214 (61.3%) were male and 6446 (38.7%) were female. An obvious seasonal pattern was observed for all reported varicella cases during 2007–2018, with two onset peaks (i.e. a total of 6114 cases and 7890 cases were reported from April to July and from October to January the next year) appeared annually. A total of 16 varicella outbreaks, which involved 724 cases were reported in CISDCP during 2007–2018, and all outbreaks occurred in primary schools (50%) or junior high schools (50%). The median duration of outbreaks was 33.5 days (range: 5–117 days).

**Fig. 2.**
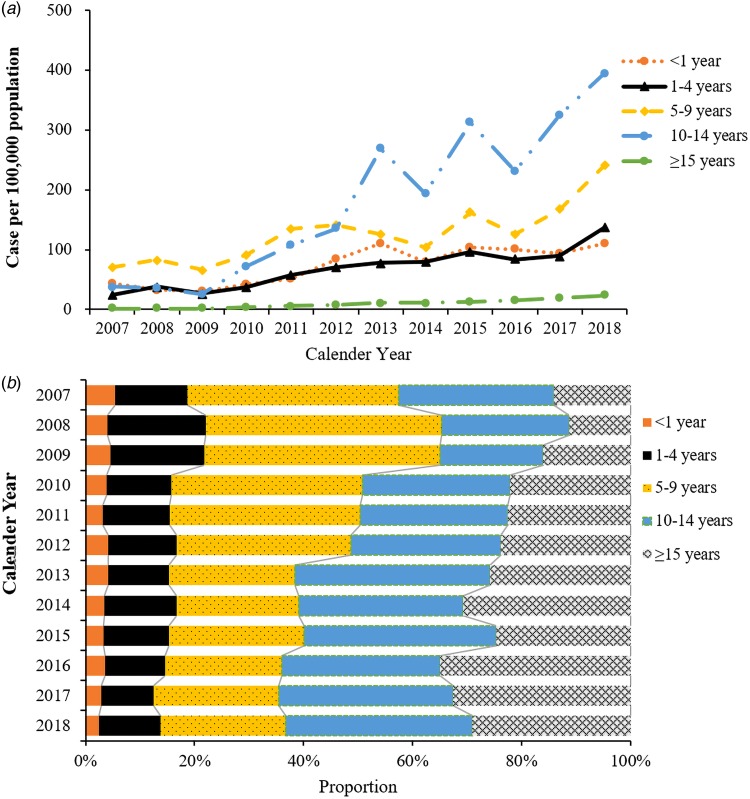
Estimated incidence (a) and proportion of cases (b) among different age groups in Lu'an, Anhui, China, 2007–2018.

Significant increases in the number of cases and estimated incidence were observed among all age groups. The annual estimated incidence was higher among 10–14 years and 5–9 years of age, with an annual incidence rate of 161 cases per 100 000 population and 126.8 cases per 100 000 population, respectively. However, a shift in age group was obviously observed since 2012, with the age increased from 5–9 years to 10–14 years (Fig. 2a). Besides, the highest increase occurred among adults more than 15 years and adolescents 10–14 years of age, with increases in incidence of 1234.6% and 942.9% from 2007 to 2018, respectively. Moreover, the proportion of reported varicella cases of adolescents aged 10–14 years and adults more than 15 years was gradually increased from 2007 to 2018 (Fig. 2b).

### Overall estimated vaccine effectiveness

A total of 432 560 children who were born between 2008 and 2017 were enrolled in the AIIMS, and 310 049 (71.7%) children had received one-dose of VarV. During the same period, 3975 varicella cases were diagnosed in children of the same birth cohort, while 686 (17.3%) cases with unknown vaccination status were excluded from the cohorts. Of the 3289 cases, 1108 (33.7%) of those cases had been vaccinated one-dose of VarV. Only one case was reported after vaccination of a second dose vaccine. As shown in Table 2, we estimated the overall VE for one-dose of VarV *via* screening method was 79.9% (95% CI: 78.3–81.2), and VE in the birth cohorts from 2008 to 2017 was estimated to range from 60.1% (95% CI: 50.3–66.2) to 96.2% (95% CI: 92.4–98.1). Besides, we observed that VE registered a progressive increase from the 2008 to 2017 birth cohorts. Compared with 2008–2012 birth cohorts, the protection afforded by one-dose of VarV in the 2013–2017 birth cohorts appears to be highly effective, and the estimated average VE was 71.1% (95% CI: 68.5–73.4) and 88.6% (95% CI: 86.9–90.1), respectively.

**Table 2.** Estimated VE for VarV by birth cohort in Lu'an, Anhui, China, 2008–2017

Birth cohort	PPV			PCV			VE, % (95% CI)
	Population	Vaccinated	%	Cases	Vaccinated	%	
2008	46 870	22 343	47.7	476	127	26.7	60.1 (50.3–66.2)
2009	45 161	28 483	63.1	551	218	39.6	61.7 (54.0–67.3)
2010	43 663	30 728	70.4	464	177	38.1	74.0 (68.4–78.3)
2011	42 866	32 589	76.0	390	160	41.0	78.1 (73.0–81.9)
2012	43 718	34 762	79.5	357	141	39.5	83.2 (79.1–86.3)
2013	40 702	31 977	78.6	293	103	35.2	85.2 (81.1–88.3)
2014	41 222	32 674	79.3	282	84	29.8	88.9 (85.6–91.3)
2015	40 821	31 157	76.3	217	61	28.1	87.9 (93.6–90.9)
2016	44 205	34 223	77.4	158	28	17.7	93.7 (90.5–95.8)
2017	43 332	31 113	71.8	101	9	8.9	96.2 (92.4–98.1)
Total	432 560	310 049	71.7	3289	1108	33.7	79.9 (78.3–81.2)

## Discussion

Live attenuated Oka strain VarV has been available in Lu'an since 2000 if a child's parent is willing to pay for it. However, information on the VC from 2000 to 2007 was missing due to exclusion in the routine immunisation and the faultiness of surveillance system. The AIIMS established in 2007 provided an excellent opportunity to evaluate the VC for VarV. The dada from the AIIMS showed that the overall VC was 71.7% (range: 47.7–79.5%) for VarV_1_ and only 1.89% for VarV_2_ (range: 0.03–3.3%) among 2008–2017 birth cohort. Furthermore, the current study indicated that there was a marked increase in reported cases in all age groups and a shift in the age group of varicella cases was obviously observed since 2012, with the age increased from 5–9 years to 10–14 years. Finally, our study demonstrated that the overall VE for VarV was moderate and wanes over time, but appears to be highly effective within 5 years after vaccination.

The WHO position is that VC that remains less than 80% over the long term is expected to shift varicella infection to older ages in some settings, which may result in an increase of morbidity and mortality [16]. Frustratingly, an upward shift in the age distribution of varicella and an increase of morbidity have been observed in Lu'an during the voluntary vaccination era over 10 years, which appears to be consistent with the finding of varicella epidemiology tendency at the national level [10]. We found that the estimated incidence increased from 10.0 cases per 100 000 population in 2007 to 65.2 cases per 100 000 population in 2018, with significant increases among all age groups, especially among subjects aged more than 15 years old and among adolescents 10–14 years of age. Besides, a shift in the age group of varicella cases was obviously observed since 2012, with the age increased from 5–9 years to 10–14 years. This shift in the age at infection may be caused by the long-term moderate level (range: 47.7–79.5%) VC of one-dose VarV. Mathematical models have demonstrated that even two-dose varicella vaccination at the intermediated levels of coverage may also lead to an increase in adult varicella [17], suggesting that a higher coverage is likely to be needed to improve disease control. Moreover, a recent study indicated that high coverage is the critical success factor among efficacy, coverage, number of doses and dosing interval [18]. Besides, our results also showed that the VC of MCV_2_ which was introduced in routine immunisation programme since 2008 in China was much higher than VarV. Therefore, to reduce varicella morbidity and prevent any potential shift to older age groups, the introduction of VarV into routine immunisation programme is likely needed in China first. Even if one-dose of VarV schedule could not be included into routine immunisation due to consideration of cost-effectiveness, an intervention measure should be implemented in regions with the long-term low or moderate level of VC to maintain a coverage of >80%.

Our study also was performed to estimate the VE for one-dose of VarV during voluntary era in Lu'an using the screening method. The results of the current study showed that the overall VE for one-dose of VarV was 79.9%, in agreement with other studies [19, 20]. Besides, we noted that VE registered a progressive increase from 60.1% in 2008 birth cohort to 96.2% in 2017 birth cohort. Although evidence on the loss of vaccine-induced protection after one-dose vaccination has been inconclusive in previous studies [21, 22], more and more studies revealed that the VE of VarV wanes over time [23–25]. Our study showed that the VE of one-dose of VarV was very high in the first 5 years and fell in children who had been vaccinated more than 6 years, which supports the role of waning immunity as an explanation of the suboptimal effectiveness of a single dose of VarV. Since vaccine-induced immunity is not lifelong, suggesting a necessitate booster dose is likely to be needed in later life [26]. However, the cost–benefit effect between one-dose and two-dose varicella vaccination should be balanced before countries decide on the introduction of a two-dose schedule into routine immunisation.

Our findings are subject to the following limitations. First, since CISDCP is a passive surveillance system, varicella cases who did not seek medical help may be not reported in it. As a result, the incidence of varicella might be underestimated in our study. On the other hand, although there was no any special public health intervention policy in Lu'an from 2007 to 2018, it is not clear that whether the increase of varicella incidence is caused by the increased awareness of doctors themselves. However, the surveillance data are still representative enough to describe the changing epidemiology of varicella in Lu'an. Second, the data for VC analysis relied on the immunisation records of children registered in AIIMS and the children who were not registered might have a lower VC. Therefore, we could have overestimated the VC for VarV. Third, the names and the date of birth of cases registered in CISDCP and AIIMS may be inconsistent, resulting in inaccurate vaccination information of varicella cases during our data collection. Thus, underestimation or overestimation of VE for VarV would not be avoided. Despite these limitations, our study provides valuable evidence to guide the varicella immunisation strategy in China.

## Conclusions

In conclusion, during the voluntary era, the overall VE for VarV was moderate and wanes over time, but appears to be highly effective within 5 years after vaccination. Our study also indicated that the situation of a shift varicella infection to older ages occurred at the long-term moderate level of VC of one-dose VarV. Continued surveillance will be important to monitor the changing epidemiology of varicella during one-dose voluntary vaccination policy. Besides, to contain the prevalence of varicella and prevent any potential shift to older ages, the introduction of VarV into routine immunisation programme is likely needed in Lu'an.
